# Intra-Species and Inter-Kingdom Signaling of *Legionella pneumophila*

**DOI:** 10.3389/fmicb.2017.00079

**Published:** 2017-02-03

**Authors:** Ramon Hochstrasser, Hubert Hilbi

**Affiliations:** Department of Medicine, Institute of Medical Microbiology, University of ZürichZürich, Switzerland

**Keywords:** α-hydroxyketone, amoeba, autoinducer, bacterial pathogenesis, cell–cell communication, *Dictyostelium*, *Legionella*, macrophage, small molecule signaling, phospho-transfer, response regulator, sensor kinase, quorum sensing

## Abstract

The ubiquitous Gram-negative bacterium *Legionella pneumophila* parasitizes environ mental amoebae and, upon inhalation, replicates in alveolar macrophages, thus causing a life-threatening pneumonia called “Legionnaires’ disease.” The opportunistic pathogen employs a bi-phasic life cycle, alternating between a replicative, non-virulent phase and a stationary, transmissive/virulent phase. *L. pneumophila* employs the Lqs (*Legionella* quorum sensing) system as a major regulator of the growth phase switch. The Lqs system comprises the autoinducer synthase LqsA, the homologous sensor kinases LqsS and LqsT, as well as a prototypic response regulator termed LqsR. These components produce, detect, and respond to the α-hydroxyketone signaling molecule LAI-1 (*Legionella* autoinducer-1, 3-hydroxypentadecane-4-one). LAI-1-mediated signal transduction through the sensor kinases converges on LqsR, which dimerizes upon phosphorylation. The Lqs system regulates the bacterial growth phase switch, pathogen-host cell interactions, motility, natural competence, filament production, and expression of a chromosomal “fitness island.” Yet, LAI-1 not only mediates bacterial intra-species signaling, but also modulates the motility of eukaryotic cells through the small GTPase Cdc42 and thus promotes inter-kingdom signaling. Taken together, the low molecular weight compound LAI-1 produced by *L. pneumophila* and sensed by the bacteria as well as by eukaryotic cells plays a major role in pathogen-host cell interactions.

## Inter-Bacterial and Inter-Kingdom Small Molecule Signaling

Bacteria communicate with each other through small diffusible organic molecules and thus coordinate their group behavior. This phenomenon is termed “quorum sensing” and triggered upon reaching a threshold concentration (the “quorum”) of the signaling molecule. Quorum sensing is mediated by various chemical classes of small molecules called “autoinducers” (Fuqua and Greenberg, 2002; Ng and Bassler, 2009; Shank and Kolter, 2009). Prominent examples of low molecular weight compounds promoting quorum sensing of Gram-negative bacteria include *N*-acyl-homoserine lactone (HSL) autoinducers, diffusible signal factor (DSF), autoinducer-2 (AI-2) and its precursor 4,5-dihydroxy-2,3-pentanedione (DPD), 2-heptyl-3-hydroxy-4-quinolone (PQS), as well as dialkylresorcinol (DAR) and α-hydroxyketone (AHK) molecules (Papenfort and Bassler, 2016).

The bacterial response to autoinducer classes is multi-faceted and versatile. The same compound can be detected by vastly different bacterial genera, e.g., AI-2 appears to be an almost universal signal (Pereira et al., 2013). Slightly modified molecules of the same chemical class can trigger distinct responses among different species of the same genus, e.g., AHK derivatives confer signaling specificity among *Vibrio* species (Ng and Bassler, 2009; Ng et al., 2010, 2011). Also, a number of chemically different quorum sensing molecules co-operate in a single organism, e.g., *Vibrio harveyi* responds to and integrates the signaling of *N*-(3-hydroxybutyryl)-HSL (HAI-1), autoinducer-2 (AI-2, a furanosyl borate diester), and (*Z*)-3-aminoundec-2-en-4-one (CAI-1; Ng and Bassler, 2009). Thus, in a complex environmental niche, such as a mixed-species biofilm, a plethora of chemical “languages” is spoken, and a single bacterial cell needs to selectively respond to distinct “idioms.”

Small molecule communication is not restricted to prokaryotes. Rather, reciprocal signaling also occurs between prokaryotes and eukaryotes, either of which can produce the corresponding low molecular weight molecule(s). This process is termed inter-kingdom signaling (Shiner et al., 2005; Pacheco and Sperandio, 2009). Prominent examples of autoinducers mediating inter-kingdom signaling are bacteria-produced AHLs, which modulate eukaryotic cell migration and chemotaxis (Karlsson et al., 2012; Holm and Vikstrom, 2014), cell death (Tateda et al., 2003; Schwarzer et al., 2012; Kravchenko et al., 2013; Valentine et al., 2013), inflammatory responses (Kravchenko et al., 2008; Kravchenko and Kaufmann, 2013), as well as plant development and immunity (Schikora et al., 2016). Furthermore, AHLs as well as AHKs are chemo-attractants for the nematode *Caenorhabditis elegans* (Werner et al., 2014), and AHKs play a role in bacteria-fungi interactions (Haack et al., 2016).

Host cell-produced inter-kingdom signaling molecules include adrenergic compounds (catecholamines), which are detected by a number of bacterial genera through the QseBC two-component system (TCS; Kendall and Sperandio, 2016). Phagocytes such as neutrophils and macrophages (Flierl et al., 2007, 2009), as well as amoebae (Coppi et al., 2002) synthesize and respond to the catecholamines adrenaline and noradrenaline. In turn, these hormones activate macrophages and thus restrict intracellular growth of, e.g., *Mycobacterium* spp. (Weatherby et al., 2003). In this review, we will highlight recent research on small molecule signaling underlying the intra-species and inter-kingdom signaling of the amoebae-resistant, opportunistic pathogen *Legionella pneumophila*.

## *Legionella pneumophila*: Environmental Niches and Human Infection

*Legionella pneumophila* is a ubiquitous Gram-negative bacterium that colonizes complex aquatic biofilm communities (Declerck, 2010; Abdel-Nour et al., 2013) and also forms single species biofilms in rich and minimal artificial media (Mampel et al., 2006; Piao et al., 2006; Pécastaings et al., 2010). In the environment, *L. pneumophila* preferentially parasitizes free-living protozoa (amoebae and ciliates), wherein the bacteria naturally replicate (Fields, 1996; Hoffmann et al., 2014b). Another possible niche of *L. pneumophila* is the intestinal tract of nematodes, e.g., *C. elegans*, which under laboratory conditions can be infected with the pathogen (Brassinga et al., 2010; Komura et al., 2010). The bacteria grow best at ambient temperatures (25–42°C) with an optimal growth temperature of around 35°C (Fields et al., 2002). However, *Legionella* spp. can persist at temperatures above 60°C in association with thermo-tolerant amoebae such as *Acanthamoeba, Naegleria, Hartmannella*, and *Vahlkampfia* spp. (Taylor et al., 2009).

Upon inhalation of *Legionella*-contaminated aerosols, the opportunistic pathogens reach the lung and replicate in alveolar macrophages, thus causing a severe pneumonia called Legionnaires’ disease (McDade et al., 1977; Horwitz and Silverstein, 1980), reviewed by (Newton et al., 2010; Hilbi et al., 2011). About half of the more than 55 *Legionella* spp. currently identified, have been associated with human disease; yet the clinically most relevant species are *L. pneumophila* and *Legionella longbeachae*. *L. pneumophila* serogroup (sg) 1 causes about 85% of all clinical cases in most parts of the world, while *L. longbeachae* accounts for about 30% of the reported cases in Australia and New Zealand (Fields et al., 2002; Newton et al., 2010).

Legionnaires’ disease mainly affects elderly or immuno-compromised persons and can spread in outbreaks comprising as many as 450 cases (Garcia-Fulgueiras et al., 2003). Since the identification of *L. pneumophila* 40 years ago, it was believed that the “accidental” pathogen is transmitted to humans only from environmental sources (Hilbi et al., 2010; Newton et al., 2010). However, after a recent outbreak of Legionnaires’ disease (Shivaji et al., 2014), the first case of a probable person-to-person transmission has been reported (Correia et al., 2016). The genome sequence of this outbreak strain revealed a phylogenetic divergence from most other outbreak-associated *L. pneumophila* sg1 strains studied (Borges et al., 2016). Of note, the strain harbors a mosaic genome carrying eight different horizontally acquired regions, some of which are also found in other *L. pneumophila* isolates.

## *Legionella pneumophila* Replicates Intracellularly in a Distinct Pathogen Vacuole

*Legionella pneumophila* is taken up by phagocytes through macropinocytosis, evades the canonical bactericidal endocytic pathway and instead forms a replication-permissive membrane-bound compartment, the LCV (Isberg et al., 2009; Hilbi and Haas, 2012). Using an evolutionarily seemingly conserved mechanism, the nascent LCV avoids fusion with lysosomes, but communicates with the endosomal, secretory, and retrograde vesicle trafficking pathways (Personnic et al., 2016) and finally associates with the endoplasmic reticulum (ER) in a tight manner (Swanson and Isberg, 1995; Lu and Clarke, 2005; Robinson and Roy, 2006).

*Legionella*-containing vacuole formation is a complex and robust process, which on the pathogen side requires as an essential virulence factor the Icm/Dot T4SS (Kubori and Nagai, 2016). The Icm/Dot T4SS is a multi-component molecular apparatus that translocates the stunning number of up to 300 different putative “effector” proteins into eukaryotic host cells (Hubber and Roy, 2010; Finsel and Hilbi, 2015). The effector proteins subvert crucial cellular processes, such as signal transduction, vesicle trafficking, motility, death pathways, gene expression, and protein production. Some of these effectors target host components implicated in antibacterial defense or membrane dynamics, including the chelator phytate (Weber et al., 2014a), small GTPases (Itzen and Goody, 2011; Sherwood and Roy, 2013; Hoffmann et al., 2014a), phosphoinositide (PI) lipids (Weber et al., 2006, 2014b; Ragaz et al., 2008; Brombacher et al., 2009; Haneburger and Hilbi, 2013; Dolinsky et al., 2014), the PI phosphatase OCRL (Weber et al., 2009), the retromer complex (Finsel et al., 2013), microtubules (Rothmeier et al., 2013; Simon et al., 2014), or the actin cytoskeleton (Franco et al., 2012; Guo et al., 2014; Michard et al., 2015). In summary, the plethora of Icm/Dot-translocated effector proteins subverts the host cell’s physiology in a highly sophisticated and customized manner to ensure intracellular survival and growth of the pathogen.

## The Bi-Phasic Life Cycle of *L. pneumophila*

*Legionella pneumophila* employs a bi-partite metabolism, where serine serves as major energy supply, while glycerol and carbohydrates like glucose are mainly fed into anabolic processes (Häuslein et al., 2016). The facultative intracellular pathogen survives and replicates in extracellular as well as intracellular niches. The transfer of the bacteria between different niches is facilitated by a bi-phasic life cycle, comprising a replicative, non-virulent phase and a transmissive, virulent phase (Molofsky and Swanson, 2004; Manske and Hilbi, 2014). The cycle is controlled by the bacterial growth phase and nutritional conditions (Byrne and Swanson, 1998), such as amino acid availability (Byrne and Swanson, 1998; Sauer et al., 2005) or fatty acid biosynthesis activity (Dalebroux et al., 2009).

In the post-exponential phase *L. pneumophila* up-regulates virulence, motility, and stress resistance, while in the exponential phase these traits are repressed, and metabolic pathways are up-regulated (Brüggemann et al., 2006; Faucher et al., 2011). The transition from exponential to post-exponential phase upon growth of the bacteria in broth is considered to reflect the transmission from the replicative to the transmissive phase in host cells. Collectively, the transmissive and virulence traits enable *L. pneumophila* to evade protozoan predators, survive in the environment as motile planktonic cells and re-establish a replicative niche in biofilms, protozoa or – perhaps – nematodes.

The master regulator of *L. pneumophila*’s bi-phasic life cycle is CsrA (carbon storage regulator A), a conserved and essential global activator of replication and repressor of transmission traits (Fettes et al., 2001; Molofsky and Swanson, 2003; Forsbach-Birk et al., 2004). Accordingly, the overproduction of CsrA in *L. pneumophila* leads to a reduction of flagellation (Fettes et al., 2001; Suzuki et al., 2006). CsrA is an RNA-binding regulatory protein, which is sequestered by the small non-coding RNAs (snRNAs) RsmY and RsmZ, thus relieving the repression of virulence and transmissive traits (Rasis and Segal, 2009; Sahr et al., 2009).

## Distribution of AHK-Based Quorum Sensing Systems

**FIGURE 1:**
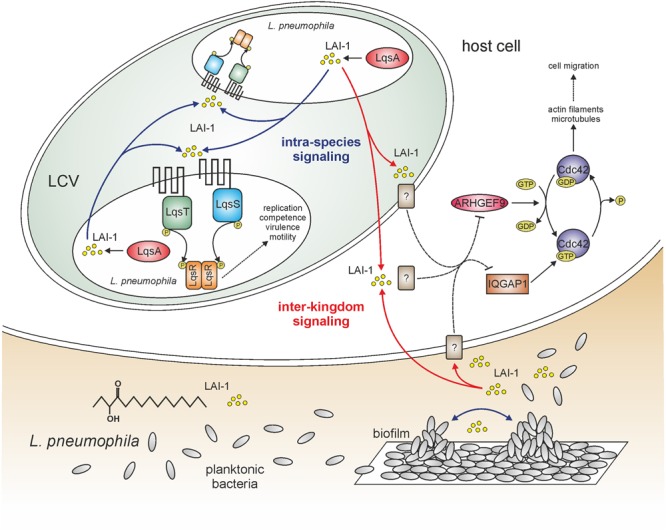
**LAI-1-mediated intra-species and inter-kingdom signaling of *Legionella pneumophila.*** The amoebae-resistant opportunistic pathogen *L. pneumophila* colonizes different niches in the environment, including biofilms and protozoa. *L. pneumophila* employs a bi-phasic life cycle, alternating between a replicative and a stationary/transmissive phase. As a major regulator of the growth phase switch *L. pneumophila* employs the Lqs system, which produces, detects, and responds to the AHK (α-hydroxyketone) signaling molecule LAI-1 (*Legionella* autoinducer-1; 3-hydroxypentadecane-4-one). The Lqs system comprises the autoinducer synthase LqsA, the sensor kinases LqsS and LqsT, as well as the response regulator LqsR. The system regulates the bacterial growth phase switch, virulence, motility, filaments, and competence. LAI-1 not only promotes bacterial cell–cell communication and quorum sensing, but also modulates the migration of eukaryotic cells through a pathway requiring the scaffold protein IQGAP1, the small GTPase Cdc42 and its activator ARHGEF9. The eukaryotic LAI-1 receptor(s) is/are unknown [?]. Solid and dashed lines represent known/direct or hypothetical/indirect pathways, respectively.

As a major regulator of the growth phase switch *L. pneumophila* employs the Lqs system (Tiaden et al., 2007), which produces, detects and responds to the AHK molecule LAI-1 (*Legionella* autoinducer-1; 3-hydroxypentadecane-4-one; Spirig et al., 2008) (**Figure 1**). The system is encoded by genes arranged in a genomic cluster (*lqsA–lqsR–hdeD–lqsS*; Tiaden et al., 2008) and an orphan gene (*lqsT*; Kessler et al., 2013). All of these genes are expressed from individual promoters (Sahr et al., 2012). *L. pneumophila* but not *L. longbeachae* harbors the Lqs system, and thus, the system is not conserved among *Legionella* spp. (Tiaden et al., 2010a). *L. pneumophila* apparently lacks AI-2- and AHL-based sensing circuits, leaving Lqs the only known quorum sensing system of this species (Tiaden et al., 2010a).

α-Hydroxyketone signaling molecules and the corresponding quorum sensing system have been first identified in *Vibrio* spp. and termed CAI-1 (cholerae autoinducer-1; 3-hydroxytridecane-4-one; Higgins et al., 2007) and CqsAS, respectively, (Miller et al., 2002; Henke and Bassler, 2004), reviewed in (Ng and Bassler, 2009; Tiaden and Hilbi, 2012). The system appears to be wide-spread, as homologs of the *cqsA* and *cqsS* genes are found not only in members of the family Legionellaceae (*Legionella* spp.) and Vibrionaceae (*Vibrio* spp., *Photobacterium* spp.), but also in the Burkholderiaceae (*Burkholderia* spp., *Cupriavidus* spp.), Chlorobiaceae (*Chlorobium* spp., *Prosthecochloris* spp.; Tiaden et al., 2010a), and Oxalobacteraceae (*Janthinobacterium* spp., *Duganella* spp.; Hornung et al., 2013; Haack et al., 2016). *Photobacterium angustum* indeed produces CqsAS orthologs and employs AHK-dependent quorum sensing (Ke et al., 2014). Given its broad distribution, AHK signaling might be commonly used for inter-species and inter-genera crosstalk among environmental bacteria.

## Functional Characterization of *L. pneumophila* Lqs System Components

Components of the *L. pneumophila* Lqs system comprise the pyridoxal-5′-phosphate-dependent autoinducer synthase LqsA (Spirig et al., 2008), the cognate membrane-bound sensor kinase LqsS (Tiaden et al., 2010b) and its homolog LqsT (Kessler et al., 2013), as well as the prototypic response regulator LqsR (**Figure 1**) (Tiaden et al., 2007). The *hdeD* gene also present in the *lqs* cluster encodes a protein of unknown function (Tiaden et al., 2008).

The biochemical characterization of LqsS and LqsT revealed that the putative sensor histidine kinases are indeed autophosphorylated by [γ-^32^P]-ATP at their conserved histidine residues (His_200_ or His_204_, respectively), located in the cytoplasmic C-terminal histidine kinase domain (Schell et al., 2014). LqsS and LqsT are both bound by LqsR or phospho-LqsR, and dependent on its conserved receiver domain aspartate (Asp_108_), the response regulator abrogated autophosphorylation of the sensor kinases by catalyzing the dephosphorylation of phospho-LqsS or phospho-LqsT. LqsR forms dimers upon phosphorylation at Asp_108_ by either acetyl-phosphate or phospho-LqsT. Upon heterologous production in *Escherichia coli* LqsT (but not LqsS) is autophosphorylated by ATP, and LqsR prevents phosphorylation of the sensor kinase under these conditions by catalyzing the dephosphorylation of phospho-LqsT. Taken together, phosphorylation signaling through the sensor histidine kinases LqsS and LqsT converges on LqsR, which forms dimers upon phosphorylation (Schell et al., 2014).

Synthetic LAI-1 inhibits autophosphorylation of LqsS or LqsT by [γ-^32^P]-ATP in a dose-dependent manner (Schell et al., 2016). LAI-1 does neither affect the stability of phospho-LqsS or phospho-LqsT, nor the dephosphorylation by LqsR, suggesting that the AHK compound inhibits the kinase reaction. In contrast, the *Vibrio cholerae* autoinducer CAI-1 (3-hydroxytridecane-4-one) promotes the phosphorylation of LqsS (but not LqsT). Moreover, synthetic LAI-1 promotes the motility of *L. pneumophila* in an LqsS/LqsT- and LqsR-dependent manner. Transcriptome analysis of *L. pneumophila* treated with LAI-1 revealed that the signaling molecule negatively regulates the RNA-binding global regulator *crsA* and positively regulates a number of genes, including the snRNAs *rsmY* and *rsmZ*. In summary, these findings indicate that LAI-1 regulates motility and the switch from the replicative to the transmissive growth phase of *L. pneumophila* by phosphorylation signaling through LqsS, LqsT and LqsR (Schell et al., 2016).

## *Legionella pneumophila* Traits Regulated by the Lqs System

To assess the function of the *lqs* genes genetically, the individual genes or the entire *lqs* gene cluster (*lqsA–lqsR–hdeD–lqsS*) were deleted from the *L. pneumophila* chromosome by double homologous recombination (Tiaden et al., 2007, 2008, 2010b; Kessler et al., 2013). These studies revealed that the Lqs system regulates a number of processes in *L. pneumophila*, including the entry into replicative growth phase, pathogen-phagocyte interactions, bacterial motility, the formation of extracellular filaments, natural competence for DNA uptake, and the expression of a *bona fide* genomic “fitness island.”

*Legionella pneumophila* lacking *lqsA* is only mildly defective for pathogen-host cell interactions (Tiaden et al., 2010b), but outcompeted by the parental strain upon co-infection of *Acanthamoeba castellanii* (Kessler et al., 2013). Moreover, the Δ*lqsA* mutant strain shows reduced motility, expression of the flagellin promoter P*_flaA_*, and flagellin production (Schell et al., 2016). Strikingly, *L. pneumophila* Δ*lqsA* takes up external DNA 3–4 orders of magnitude more efficiently, and the expression of the P*_comEA_* promoter is up-regulated. The promoter controls the expression of *comEA* encoding a small periplasmic DNA-binding protein essential for competence (Charpentier et al., 2011). These results revealed that the Lqs system is a major negative regulator of natural competence of *L. pneumophila*.

*Legionella pneumophila* lacking *lqsS* is severely defective for intracellular replication (Tiaden et al., 2010b), impaired for motility (Schell et al., 2016), outcompeted by the parental strain upon co-infection of amoebae and, similar to the Δ*lqsA* strain, more competent for DNA uptake (Kessler et al., 2013). *L. pneumophila* Δ*lqsS* also sediments slower than wild-type or Δ*lqsA* mutant bacteria, due to the formation of extracellular filaments (Tiaden et al., 2010b). Finally, in the Δ*lqsS* mutant strain 52 genes located in a 133 kb high plasticity genomic “fitness island” are up-regulated at least two-fold (Tiaden et al., 2010b). The fitness island (*lpg0973*-*lpg1096*) shows a higher G+C content than the *L. pneumophila* core genome, is located adjacent to the tRNA^Thr^ gene *lpg0972* and flanked by putative DNA-mobilizing genes such as integrases, transposases, and phage-like genes. Two regions can be discriminated: region I (*lpg0973*-*lpg1003*, 26 kb) harbors many (conserved) unknown genes, some of which encode putative pili components (PilE, PilT), and region II (*lpg1006*-*lpg1096*, 107 kb) encodes the subunits of a F_o_F_1_ ATP synthase and several metal ion resistance transporters. The region *lpg1008*-*lpg1035* has been characterized previously as a 40 kb eﬄux pump genomic island, which is induced upon (but not required for) *L. pneumophila* infection of macrophages (McClain et al., 1996; Rankin et al., 2002). Taken together, the *L. pneumophila* 133 kb genomic region fulfills the criteria of a canonical genomic “fitness island” (Dobrindt et al., 2004).

*Legionella pneumophila* lacking *lqsT* (or both *lqs* sensor kinase genes) is also severely defective for intracellular replication and outcompeted by the parental strain upon co-infection of amoebae (Kessler et al., 2013), impaired for motility (Schell et al., 2016), as well as – similar to the Δ*lqsS* and Δ*lqsA* strains – more competent for DNA uptake (Kessler et al., 2013). However, in contrast to *lqsS, lqsT* does not regulate the production of extracellular filaments. The *lqsT* and *lqsS* genes are divergently expressed in the post-exponential growth phase, and transcriptome studies reveal that 90% of the genes down-regulated in absence of *lqsT*, are up-regulated in absence of *lqsS*. Reciprocally regulated genes encode constituents of the 133 kb genomic island or Icm/Dot-translocated effectors. The phenotypes of a mutant strain lacking *lqsS* and *lqsT* are partially complemented by either *lqsT* or *lqsS*, but are not reversed by overexpression of *lqsA*, as the single mutants are. This suggests that LqsT and LqsS are the sole LAI-1-responsive sensor kinases in *L. pneumophila*. Collectively, these results indicate that the Lqs system comprises two partially antagonistic LAI-1-responsive sensor kinases, which regulate distinct pools of genes implicated in various physiological and pathogenic processes of *L. pneumophila*.

*Legionella pneumophila* lacking *lqsR* shows a reduced lag phase before initiating growth in broth, and thus, the response regulator LqsR regulates the switch between the transmissive and the replicative phase (Tiaden et al., 2007). The Δ*lqsR* mutant strain is defective for uptake by and intracellular replication in phagocytes (Tiaden et al., 2007), impaired for motility (Schell et al., 2016), outcompeted by the parental strain upon co-infection of amoebae, and more competent for DNA uptake (Kessler et al., 2013). The virulence phenotypes of Δ*lqsR* are stronger than those of the other *lqs* single mutant strains. Yet, *L. pneumophila* lacking the entire *lqs* cluster showed even more severe and pleiotropic phenotypes, suggesting that the *lqs* genes act synergistically (Tiaden et al., 2008).

In summary, these studies revealed a unique organization of the *L. pneumophila* Lqs system comprising two homologous, partially antagonistic LAI-1-responsive sensor kinases. The Lqs system and LAI-1 circuit regulate many distinct features of *L. pneumophila*, in particular transmissive traits, such as pathogen-host cell interactions and virulence, bacterial motility, natural competence, production of extracellular filaments, and expression of a genomic “fitness island.” Hence, the Lqs system is a major regulator of the bi-phasic life cycle of *L. pneumophila*.

## Connection of the Lqs System With Other *L. pneumophila* Two-Component Systems

**FIGURE 2:**
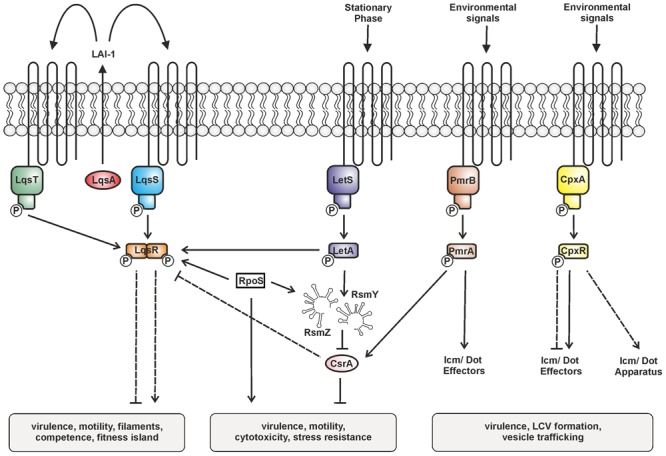
**Two-component systems (TCSs) controlling the bi-phasic life cycle of *L. pneumophila*.** The TCSs LqsRS, LetAS, PmrAB, and CpxRA, as well as the RsmYZ-CsrA regulatory unit are illustrated. Under conditions of nutrient abundance the RNA-binding carbon storage regulator A (CsrA) represses transmission traits and promotes replication. Upon nutrient depletion (at the onset of stationary phase), LetAS and the sigma factor RpoS up-regulate transmission traits. LetA induces the small non-coding snRNAs RsmY and RsmZ, which sequester CsrA and relieve post-transcriptional repression. LAI-1 produced by LqsA is detected by and prevents phosphorylation of the sensor kinases LqsS and LqsT. Thus, the cognate response regulator LqsR is dephosphorylated. LqsR is positively regulated by RpoS and negatively regulated by the LetA-RsmYZ-CsrA cascade. The response regulators PmrA and CpxR regulate the production of Icm/Dot components and substrates. The signals triggering the sensor histidine kinases LetS, PmrB, and CpxA are not known. Solid and dashed lines represent known/direct or hypothetical/indirect regulation, respectively.

The production of LqsR is controlled by the stationary phase sigma factor RpoS, and, less stringently, by the response regulator LetA (Tiaden et al., 2007), as well as on a post-transcriptional level probably by CsrA (Sahr et al., 2009). Thus, the Lqs system represents an element of the stationary growth phase virulence regulatory network of *L. pneumophila* comprising several TCSs (**Figure 2**) (Segal, 2013). The network includes the LetAS TCS, which is homologous to *Pseudomonas* GacAS (Laville et al., 1992). Upon entering stationary phase, LetAS regulates *L. pneumophila* transmission and virulence by promoting motility, contact-dependent cytotoxicity, infectivity, and evasion of lysosomes in macrophages (Hammer et al., 2002), as well as intracellular replication in *A. castellanii* (Gal-Mor and Segal, 2003b; Lynch et al., 2003). The response regulator LetA, in concert with RpoS, directly up-regulates the snRNAs *rsmY* and *rsmZ*, which bind and sequester the global repressor CsrA (Rasis and Segal, 2009; Sahr et al., 2009).

The PmrAB TCS is a direct regulator of several Icm/Dot-translocated effector proteins and required for motility and intracellular replication of *L. pneumophila* in protozoa and macrophages (Zusman et al., 2007; Al-Khodor et al., 2009). The response regulator PmrA positively regulates metabolic genes including *csrA* and thus links the TCS with the LetA-RsmYZ-CsrA regulatory cascade (Rasis and Segal, 2009).

Finally, the CpxRA TCS plays a major role in *L. pneumophila* virulence gene regulation by controlling the expression of the *icmR* gene and at least 27 Icm/Dot substrates, as well as type II-secreted virulence factors (Gal-Mor and Segal, 2003a; Altman and Segal, 2008; Feldheim et al., 2016; Tanner et al., 2016). CpxR is a dual regulator, which acts as an activator or repressor, and can still self-interact in absence of phosphorylation to repress but not activate target genes (Feldheim et al., 2016).

## Inter-Kingdom Signaling Through Lai-1

Small signaling molecules promote the communication not only between bacteria but also between prokaryotes and eukaryotes. To test the hypothesis that the Lqs system and LAI-1 affect eukaryotic cells, we used migration of eukaryotic cells as readout. *L. pneumophila* inhibits the chemotactic migration of amoebae, macrophages, and neutrophils in an Icm/Dot-dependent manner (Simon et al., 2014). Rather unexpectedly, however, *L. pneumophila* lacking *lqsA* no longer impeded the migration of infected amoebae or macrophages, and the defect was complemented by plasmid-borne *lqsA* (Simon et al., 2015). Interestingly, the overexpression of *lqsA* in an Icm/Dot deficient Δ*icmT* mutant strain abolished cell migration, indicating that the overproduction of LqsA and in consequence LAI-1 inhibits cell migration (in parallel to the Icm/Dot T4SS). In agreement with this notion, synthetic LAI-1 (as well as the *Vibrio* autoinducer CAI-1) dose-dependently inhibited cell migration (**Figure 1**). That is, LAI-1 reduced the forward migration index but not the velocity, suggesting that the directionality but not speed of the cells was affected.

In order to determine host cell factors implicated in LAI-1-dependent cell migration inhibition, a scratch wound healing assay with A549 lung epithelial cells was used, alongside RNA interference (Simon et al., 2015). The depletion of any host factor relevant for LAI-1-dependent inter-kingdom signaling will abrogate the effects of synthetic LAI-1. This approach revealed that LAI-1-dependent inhibition of cell migration involves the scaffold protein IQGAP1, the small GTPase Cdc42 and the Cdc42-specific guanine nucleotide exchange factor (GEF) ARHGEF9 (**Figure 1**), but not other modulators of Cdc42, or the small GTPases RhoA, Rac1 or Ran. Furthermore, upon treatment with LAI-1, IQGAP1 redistributed to the cell cortex, and Cdc42 was inactivated. Taken together, the results indicate that LAI-1 modulates migration of eukaryotic cells through a signaling pathway involving IQGAP1, Cdc42, and ARHGEF9. The eukaryotic receptor(s) of *L. pneumophila* LAI-1 is/are currently not known.

Interestingly, *Salmonella enterica* serovar Typhimurium also modulates the actin cytoskeleton through Cdc42 and IQGAP1. The *Salmonella* type III-secreted GEF SopE directly activates Cdc42 (Hardt et al., 1998), and bacterial invasion of fibroblasts lacking IQGAP1 was decreased, while Cdc42 and Rac1 activation was abrogated (Brown et al., 2007).

## Effects of Adrenergic Antagonists on *L. pneumophila*

Inter-kingdom communication not only occurs from prokaryotes to eukaryotes but also in a reciprocal manner. A prominent example of the latter is adrenergic signaling mediated by the catecholamines adrenaline (epinephrine) and noradrenaline (norepinephrine) produced by eukaryotic cells (Hughes and Sperandio, 2008). The adrenergic neurotransmitters adrenaline and noradrenaline moderate eukaryotic stress response, but are also agonists of virulence and motility gene expression of enterohemorrhagic *Escherichia coli* (EHEC) O157:H7 (Sperandio et al., 2003; Clarke et al., 2006; Njoroge and Sperandio, 2012). Accordingly, α- and β-adrenergic antagonists (targeting the α- or β-subgroup of adrenergic receptors) block the response of EHEC to these host hormones (Hughes and Sperandio, 2008).

Bacteria respond to adrenergic compounds through TCSs, in particular QseBC and QseEF (Kendall and Sperandio, 2016). The sensor kinase QseC has been identified as adrenergic receptor in EHEC (Clarke et al., 2006) as well as in uropathogenic *Escherichia coli* (UPEC; Kostakioti et al., 2009), and EHEC also employs QseE as a receptor (Reading et al., 2009). In *S. enterica* serovar Typhi the responses to adrenergic signaling appear to depend on the CpxRA TCS (Karavolos et al., 2011). The QseBC TCS is wide-spread among bacteria, including Enterobacteriaceae, *Pseudomonas aeruginosa*, and *L. pneumophila* (Rasko et al., 2008).

The genes apparently encoding the QseBC homolog in *Legionella* spp. have been named either *pmrAB, qseBC*, or *lrpR*, depending on the strain or species involved, and adrenergic compounds were assessed for effects on *Legionella*-phagocyte interactions (Harrison et al., 2015b). Adrenaline and noradrenaline had only mild, if any, effects on *L. pneumophila* growing in broth or intracellularly in phagocytes. However, the adrenergic receptor antagonists benoxathian, naftopidil, propranolol, and labetalol reduced the growth of *L. pneumophila* in broth or amoebae, while replication in macrophages was enhanced (Harrison et al., 2015b). Growth restriction was common to several members of the genus *Legionella* and also observed for *Mycobacterium* spp. The deletion of *L. pneumophila pmrAB* (*qseBC*) had only a minor effect on growth inhibition by adrenergic antagonists, implying a different mode of action and/or the presence of another adrenergic sensing system. Yet, regardless of their bacterial target, adrenergic antagonists might represent potential lead compounds in screens for novel anti-infective compounds against *Legionella* or *Mycobacterium* spp. using phagocytes as host cells (Harrison et al., 2013, 2015a; Kicka et al., 2014).

## Conclusions

The causative agent of Legionnaires’ disease, *L. pneumophila*, is an amoebae-resistant opportunistic pathogen, which employs the AHK molecule LAI-1 for intra-species as well as inter-kingdom communication. Since AHK molecules are wide-spread in nature, environmental bacteria likely employ this class of signals for inter-genera signaling. *L. pneumophila* produces, detects and responds to LAI-1 through the Lqs system, which comprises the autoinducer synthase LqsA, the homologous sensor kinases LqsS and LqsT, as well as the response regulator LqsR. As part of the stationary phase regulatory network, the Lqs system regulates the growth phase switch, pathogen-host cell interactions, bacterial motility, natural competence, filament production and expression of a chromosomal “fitness island.” The responses of *L. pneumophila* to LAI-1 might be exploited for anti-virulence drug development, as has been described for *Vibrio cholerae* using agonist and antagonist derivatives of the corresponding signaling molecule CAI-1 (Bolitho et al., 2011). Furthermore, LAI-1 modulates the migration direction of eukaryotic cells and thus mediates inter-kingdom signaling. Adrenergic compounds synthesized by eukaryotic cells might also be sensed by *L. pneumophila*; however, the mere fact that the pathogen responds to adrenergic antagonists does not imply that this is the case. Future studies will address the question whether there are other classes of low molecular weight molecules produced and detected by *Legionella* spp. or by *Legionella* -infected host cells, and will explore the potential of small molecule signaling to interfere with *Legionella* virulence or eukaryote processes.

## Author Contributions

RH and HH wrote the manuscript.

## Conflict of Interest Statement

The authors declare that the research was conducted in the absence of any commercial or financial relationships that could be construed as a potential conflict of interest.

## Abbreviations

CAI-1: cholerae autoinducer-1
*cqs*: cholerae quorum sensing
*icm*/*dot*: intracellular multiplication/defective organelle trafficking
LAI-1: *Legionella* autoinducer-1
LCV: *Legionella*-containing vacuole
*lqs*: *Legionella* quorum sensing
T4SS: type IV secretion system
